# Supplementary material for: Comparison of the triglyceride-glucose index and triglyceride-glucose-body mass index for predicting non-alcoholic fatty liver disease in elderly diabetic patients
Source: PLoS One. 2026 Feb 2;21(2):e0341109. doi: 10.1371/journal.pone.0341109 (PMC12863506; doi:10.1371/journal.pone.0341109)
Supplement: S1 Table — (DOCX) [file pone.0341109.s001.docx]

Supplemental Table S1 The baseline characteristics of the training and validation groups in diabetes population.

|  |  |  |  |  |
| --- | --- | --- | --- | --- |
|  | All | Training Group | Validation Group | P-value |
| N | 6882 | 4817 | 2065 |  |
| age (mean (SD)) |  | 71.32 (7.05) | 71.43 (7.08) | 0.55 |
| gender， Male (%) | 3633(52.7) | 2544 (52.8) | 1089 (52.7) | 0.974 |
| BMI (Kg/㎡, mean (SD)) |  | 25.14 (36.57) | 24.56 (3.22) | 0.472 |
| SBP (mmHg, mean (SD)) |  | 144.58 (158.95) | 141.38 (18.10) | 0.361 |
| DBP (mmHg, mean (SD)) |  | 78.42 (9.60) | 78.31 (9.68) | 0.678 |
| HR (bpm, mean (SD)) |  | 73.67 (11.69) | 73.72 (11.83) | 0.852 |
| Hypertension, Yes (%) |  | 3933 (81.6) | 1666 (80.7) | 0.361 |
| NAFLD,Yes(%) | 3137(45.6) | 2210 (45.9) | 927 (44.9) | 0.467 |
| Neutrophil (10^9/L, mean (SD)) |  | 3.75 (1.23) | 3.75 (1.23) | 0.805 |
| Lymphocyte (10^9/L, mean (SD)) |  | 2.04 (0.65) | 2.05 (0.66) | 0.611 |
| Hb (g/L,mean (SD)) |  | 139.07 (14.76) | 138.89 (14.72) | 0.632 |
| Plt(10^12/L, mean (SD)) |  | 203.17 (55.71) | 202.51 (57.65) | 0.653 |
| ALT (U/L, mean (SD)) |  | 24.43 (22.97) | 23.99 (15.51) | 0.435 |
| AST (U/L, mean (SD)) |  | 28.07 (22.59) | 27.88 (13.72) | 0.716 |
| FBG (mmol/L, mean (SD)) |  | 7.21 (2.18) | 7.19 (2.08) | 0.703 |
| UA (μmol/L, mean (SD)) |  | 342.92 (91.17) | 341.23 (89.69) | 0.479 |
| Urea nitrogen (mmol/L, mean (SD)) |  | 5.51 (1.78) | 5.54 (1.71) | 0.529 |
| Creatinine (μmol/L, mean (SD)) |  | 77.31 (23.05) | 77.75 (26.98) | 0.494 |
| Triglyceride (mmol/L, mean (SD)) |  | 1.75 (1.15) | 1.74 (1.21) | 0.767 |
| Total Cholestrol (mmol/L, mean (SD)) |  | 4.62 (1.22) | 4.66 (1.26) | 0.179 |
| HDL-C (mmol/L, mean (SD)) |  | 1.66 (0.49) | 1.67 (0.48) | 0.289 |
| LDL-C (mmol/L, mean (SD)) |  | 2.65 (0.95) | 2.71 (0.99) | 0.043 |
| TyG (mean (SD)) |  | 9.03 (0.62) | 9.02 (0.62) | 0.43 |
| TyG-BMI (mean (SD)) |  | 227.52 (328.28) | 222.01 (35.79) | 0.446 |

^[[1]](#footnote-1)^

1. SD= standard deviation, BMI= body mass index, SBP= systolic blood pressure, DBP= diastolic blood pressure, HR= heart rate, NAFLD= non-alcoholic fatty liver disease, Hb= hemoglobin, Plt= platelet count, ALT= alanine aminotransferase, AST= aspartate aminotransferase, FBG= fasting blood glucose, UA= uric acid, HDL-C= high-density lipoprotein cholesterol, LDL-C= low-density lipoprotein cholesterol, TyG= triglyceride-glucose, TyG-BMI= triglyceride-glucose-body mass index [↑](#footnote-ref-1)
