# Supplementary material for: Comparison of the triglyceride-glucose index and triglyceride-glucose-body mass index for predicting non-alcoholic fatty liver disease in elderly diabetic patients
Source: PLoS One. 2026 Feb 2;21(2):e0341109. doi: 10.1371/journal.pone.0341109 (PMC12863506; doi:10.1371/journal.pone.0341109)
Supplement: S2 Table — (DOCX) [file pone.0341109.s002.docx]

**Supplemental Table S2.** **The prevalence of NAFLD in different age groups in the training and validation groups.**

| Gender | Age group | Training Group | | | | | Validation Group | | | | |
| --- | --- | --- | --- | --- | --- | --- | --- | --- | --- | --- | --- |
|  |  | Non-NAFLD | | NAFLD | | P-value | Non-NAFLD | | NAFLD | | P-value |
| Male | 60-69 | 549 | 50.46% | 539 | 49.54% | <0.001 | 245 | 53.49% | 213 | 46.51% | 0.004 |
|  | 70-79 | 486 | 55.48% | 390 | 44.52% |  | 206 | 52.96% | 183 | 47.04% |  |
|  | >80 | 221 | 71.52% | 88 | 28.48% |  | 89 | 68.99% | 40 | 31.01% |  |
| Female | 60-69 | 555 | 50.09% | 553 | 49.91% | <0.001 | 242 | 50.00% | 242 | 50.00% | <0.001 |
|  | 70-79 | 510 | 49.37% | 523 | 50.63% |  | 237 | 55.63% | 189 | 44.37% |  |
|  | >80 | 286 | 70.97% | 117 | 29.03% |  | 119 | 66.48% | 60 | 33.52% |  |
| Total | 60-69 | 1104 | 50.27% | 1092 | 49.73% | <0.001 | 487 | 51.70% | 455 | 48.30% | <0.001 |
|  | 70-79 | 996 | 52.17% | 913 | 47.83% |  | 443 | 54.36% | 372 | 45.64% |  |
|  | >80 | 507 | 71.21% | 205 | 28.79% |  | 208 | 67.53% | 100 | 32.47% |  |
