# Supplementary material for: Comparison of the triglyceride-glucose index and triglyceride-glucose-body mass index for predicting non-alcoholic fatty liver disease in elderly diabetic patients
Source: PLoS One. 2026 Feb 2;21(2):e0341109. doi: 10.1371/journal.pone.0341109 (PMC12863506; doi:10.1371/journal.pone.0341109)
Supplement: Attachment — Submitted filename: Review of Article-20250425.docx [file pone.0341109.s003.docx]

**Summary:**

In this cross-sectional study, the author evaluated the effectiveness of the triglyceride-glucose index (TyG) and triglyceride-glucose-body mass index (TyG-BMI) in identifying non-alcoholic fatty liver disease (NAFLD) through a clinical study containing 6,882 elderly patients with diabetes mellitus (DM). Participants were divided into training and validation groups to assess diagnostic performance. Both TyG and TyG-BMI were significantly associated with NAFLD risk, with TyG-BMI showing believable predictive accuracy. In addition, Receiver-operating characteristic curve (AUROC) and Decision Curve Analysis (DCA) confirmed TyG-BMI as a more reliable clinical marker. Two TyG-BMI cutoffs were proposed: <212.886 to rule out NAFLD (sensitivity ~80%, NPV ~77%) and ≥251.741 to rule in NAFLD (specificity ~91%, PPV ~76%), consistent across both groups. Thus, these findings suggest TyG-BMI is a simple, non-invasive, and cost-effective method for NAFLD detection and classification in elderly diabetic populations. However, several issues still need to be addressed.

**Comments:**

1. In line 47-28, "when steatosis is less than 20% or in morbidly obese individuals", I think "especially when hepatic fat infiltration is below 20% or in cases of morbid obesity." is more suitable.
2. In line 61-62, "this demographic" could be better to specify "elderly patients with diabetes" again for clarity.
3. The author needs to carefully check their manuscript, for example, line 136, 213, 214, the Error! Reference source not found. And The figure legends should be organized well in a divided part.
4. The selected participants who had undergone liver ultrasound. This could introduce selection bias, because people undergoing liver ultrasound might already have suspected liver/metabolic issues.
5. The author defines DM as FBG >7.0 mmol/L or history of DM. However, this excludes patients diagnosed by HbA1c ≥6.5% or 2-hour OGTT ≥11.1 mmol/L according to ADA guidelines.
6. In line 66, "cross-section study" should be "cross-sectional study"; In line 66 "waved" should be “waived”; In line 79, "flowchart of the selection of participants are shown" should be "is shown".
