# Supplementary material for: Comparison of the triglyceride-glucose index and triglyceride-glucose-body mass index for predicting non-alcoholic fatty liver disease in elderly diabetic patients
Source: PLoS One. 2026 Feb 2;21(2):e0341109. doi: 10.1371/journal.pone.0341109 (PMC12863506; doi:10.1371/journal.pone.0341109)
Supplement: Attachment — Submitted filename: PLOS ONE Review Report.docx [file pone.0341109.s004.docx]

**PLOS ONE Review Report**

Manuscript Title: Comparison of the triglyceride-glucose index and triglyceride-glucose-body mass index for predicting non-alcoholic fatty liver disease in elderly diabetic patients

Dear Dr. Qian Wu,

Thank you for inviting me to review this manuscript. I have completed the peer review process, and my detailed comments are summarized below.

**Overall Evaluation**

This study utilized a cross-sectional design to compare the predictive efficacy of the TyG index and TyG-BMI index for non-alcoholic fatty liver disease (NAFLD) in elderly diabetic patients. With a relatively large sample size (n=6,882) and methodologically sound design, the findings suggest that the TyG-BMI index outperforms the TyG index in clinical utility. The research topic aligns with the current demand for non-invasive diagnostic tools. However, several methodological details require further clarification, and the discussion could benefit from deeper exploration.

**Recommendations**

1. Limitations of Ultrasound for NAFLD Diagnosis

The lower sensitivity of ultrasound may lead to potential underdiagnosis of NAFLD cases. It is recommended to supplement the Discussion section with an acknowledgment of this bias and its impact on the study’s conclusions.

2. Biological Interpretation of TyG-BMI

The cutoff values for TyG-BMI (212.886 and 251.741) should be contextualized within clinical frameworks. Comparisons with cutoffs from prior studies (e.g., Huet al.) are encouraged to elucidate discrepancies and explore underlying reasons.

3. Control of Confounding Factors

While Model 3 adjusted for liver enzymes (AST/ALT), the manuscript omits mention of other metabolic markers (e.g., HbA1c, insulin levels). Please clarify whether these variables were assessed for potential confounding effects or discuss limitations accordingly.

4. Depth of Discussion

Strengthen the Discussion by addressing the clinical implications and translational significance of the findings. Examples include:

- Is the TyG-BMI cutoff applicable to other ethnicities or regions?

- How can this tool be integrated into existing NAFLD screening workflows (e.g., as a replacement for or complement to ultrasound)?

5. Enrichment of Conclusions

Emphasize scenarios where the TyG-BMI index holds particular promise for implementation, such as primary care screening, to underscore its clinical relevance.

6. Ethics Statement Clarification

The ethics declaration cites "previous data and cross-sectional design" to justify exemption from informed consent. However, the degree of data anonymization and original consent scope for future research use remain unclear. It is recommended to supplement whether the data is completely anonymous (cannot be traced back to individuals), and to confirm whether the original data collection included consent clauses for future research purposes.

7. English Grammar

Enhance grammatical accuracy throughout the manuscript. For example, the lines 28-29 in the abstract (The AUCs of TyG and TyG-BMI indicated that both had predictive value for NAFLD, with TyG-BMI showing the highest predictive accuracy) contains a superlative error. As only two indices are compared, "higher" is more appropriate than "highest."

Thank you for your invitation again.

Sincerely,

Yang Zhang
