# Supplementary material for: Comparison of the triglyceride-glucose index and triglyceride-glucose-body mass index for predicting non-alcoholic fatty liver disease in elderly diabetic patients
Source: PLoS One. 2026 Feb 2;21(2):e0341109. doi: 10.1371/journal.pone.0341109 (PMC12863506; doi:10.1371/journal.pone.0341109)
Supplement: Attachment — Submitted filename: Response to Reviewers.docx [file pone.0341109.s007.docx]

Reviewer #1: The authors need to carefully reorganize the manuscript, as there are numerous formatting mistakes throughout. However, the quality of the data presentation and the validity of the conclusions are sufficient for publication. Please revise the manuscript according to the comments provided in the attached file.

**Summary:**

In this cross-sectional study, the author evaluated the effectiveness of the triglyceride-glucose index (TyG) and triglyceride-glucose-body mass index (TyG-BMI) in identifying non-alcoholic fatty liver disease (NAFLD) through a clinical study containing 6,882 elderly patients with diabetes mellitus (DM). Participants were divided into training and validation groups to assess diagnostic performance. Both TyG and TyG-BMI were significantly associated with NAFLD risk, with TyG-BMI showing believable predictive accuracy. In addition, Receiver-operating characteristic curve (AUROC) and Decision Curve Analysis (DCA) confirmed TyG-BMI as a more reliable clinical marker. Two TyG-BMI cutoffs were proposed: <212.886 to rule out NAFLD (sensitivity ~80%, NPV ~77%) and ≥251.741 to rule in NAFLD (specificity ~91%, PPV ~76%), consistent across both groups. Thus, these findings suggest TyG-BMI is a simple, non-invasive, and cost-effective method for NAFLD detection and classification in elderly diabetic populations. However, several issues still need to be addressed.

**Comments:**

1. In line 47-28, "when steatosis is less than 20% or in morbidly obese individuals", I think "especially when hepatic fat infiltration is below 20% or in cases of morbid obesity." is more suitable.

Answer: Thank you for your valuable suggestion. We have revised the sentence accordingly to improve clarity and readability. The updated sentence now reads:"Moreover, ultrasound may fail to detect hepatic steatosis, especially when hepatic fat infiltration is below 20% or in cases of morbid obesity." in Line 47-48.

2. In line 61-62, "this demographic" could be better to specify "elderly patients with diabetes" again for clarity.

Answer: Thank you for your insightful comment. To enhance clarity, we have revised the phrase “this demographic” to “elderly patients with diabetes” in Line 63-64 as suggested. We appreciate your careful review.

3. The author needs to carefully check their manuscript, for example, line 136, 213, 214, the Error! Reference source not found. And The figure legends should be organized well in a divided part.

Answer: Thank you for pointing this out. We have carefully reviewed the manuscript and removed all instances of “Error! Reference source not found.” including those in lines 136, 213, and 214. Additionally, the figure legends have been reorganized and clearly divided to improve clarity. We appreciate your helpful feedback.

4. The selected participants who had undergone liver ultrasound. This could introduce selection bias, because people undergoing liver ultrasound might already have suspected liver/metabolic issues.

Answer: Thank you for your insightful comment. We understand the concern regarding potential selection bias due to the inclusion of participants who underwent liver ultrasonography. However, we would like to clarify that the study population was derived from a large cohort of individuals undergoing routine physical examinations, not limited to those with known or suspected liver or metabolic diseases. Therefore, the included participants consisted of both individuals with potential liver abnormalities and those without any known issues. This approach helps reduce the likelihood of significant selection bias and enhances the generalizability of our findings.

5. The author defines DM as FBG >7.0 mmol/L or history of DM. However, this excludes patients diagnosed by HbA1c ≥6.5% or 2-hour OGTT ≥11.1 mmol/L according to ADA guidelines.

Answer: Thank you for your thoughtful comment. We acknowledge that the American Diabetes Association (ADA) guidelines include additional diagnostic criteria for diabetes mellitus (DM), such as HbA1c ≥6.5% or 2-hour plasma glucose ≥11.1 mmol/L following an oral glucose tolerance test (OGTT). However, in this retrospective study, we followed the approach used in several previous studies, where DM was defined based on fasting blood glucose (FBG) >7.0 mmol/L or a documented history of diabetes. For example, similar definitions were applied in the studies by Hu et al. (2022) ^[1]^ and Wang et al. (2021) ^[2]^, which focused on large-scale population-based screening for NAFLD and related metabolic disorders.

References:

1.Hu H, Han Y, Cao C, He Y. The triglyceride glucose-body mass index: a non-invasive index that identifies non-alcoholic fatty liver disease in the general Japanese population. J Transl Med. 2022;20(1):398.

2.Wang R, Dai L, Zhong Y, Xie G. Usefulness of the triglyceride glucose-body mass index in evaluating nonalcoholic fatty liver disease: insights from a general population. Lipids Health Dis. 2021;20(1):77.

6. In line 66, "cross-section study" should be "cross-sectional study"; In line 66 "waved" should be “waived”; In line 79, "flowchart of the selection of participants are shown" should be "is shown".

Answer: Thank you for carefully reviewing the manuscript and identifying these language errors. We have corrected “cross-section study” to “cross-sectional study”, and revised the sentence to “the flowchart of the selection of participants is shown” as suggested. “waved” was deleted and “the Ethics Committee granted a waiver of informed consent” was applied instead. We appreciate your attention to detail.

Reviewer #2: Overall Evaluation

This study utilized a cross-sectional design to compare the predictive efficacy of the TyG index and TyG-BMI index for non-alcoholic fatty liver disease (NAFLD) in elderly diabetic patients. With a relatively large sample size (n=6,882) and methodologically sound design, the findings suggest that the TyG-BMI index outperforms the TyG index in clinical utility. The research topic aligns with the current demand for non-invasive diagnostic tools. However, several methodological details require further clarification, and the discussion could benefit from deeper exploration.

Recommendations

**1. Limitations of Ultrasound for NAFLD Diagnosis
The lower sensitivity of ultrasound may lead to potential underdiagnosis of NAFLD cases. It is recommended to supplement the Discussion section with an acknowledgment of this bias and its impact on the study’s conclusions.**

Answer: We appreciate the reviewer’s insightful comment. In response, we have revised the Discussion section to acknowledge the potential limitations of using ultrasound for NAFLD diagnosis. Specifically, we added the following statement:
"Although ultrasound examination has been widely used for diagnosing NAFLD, its relatively low sensitivity may lead to underdiagnosis of NAFLD cases. This limitation could introduce selection bias or information bias into the study, thereby affecting the accuracy and reliability of the results." highlighted in Line 293-294
This addition aims to clarify the potential impact of diagnostic limitations on our findings and to enhance the transparency of our study.

**2. Biological Interpretation of TyG-BMI**
 **The cutoff values for TyG-BMI (212.886 and 251.741) should be contextualized within clinical frameworks. Comparisons with cutoffs from prior studies (e.g., Huet al.) are encouraged to elucidate discrepancies and explore underlying reasons.**

Answer: Thank you for your valuable suggestion. We have compared our TyG-BMI cutoffs (212.886 and 251.741) with those from prior studies (e.g., Hu et al and Wang el al.) in the revised manuscript. We have analyzed potential discrepancies and explore underlying reasons in the Discussion section.

*Hu et al. previously demonstrated in a Japanese cohort that two TyG-BMI cutoff values (182.2 and 224.0) could accurately identify NAFLD and reduce unnecessary ultrasonography in the population [(4)]. Xuejie Wang et al. further determined the optimal TyG-BMI cutoff value of 218.08 in a Chinese general population. Notably, while the cutoff values in our study were higher than those reported in the Japanese population, they showed similarity to the thresholds established in Chinese populations. (View in Discussion section in Iine 268-273 )*

**3. Control of Confounding Factors
While Model 3 adjusted for liver enzymes (AST/ALT), the manuscript omits mention of other metabolic markers (e.g., HbA1c, insulin levels). Please clarify whether these variables were assessed for potential confounding effects or discuss limitations accordingly.**

Answer: Thank you for your critical insight regarding the inclusion of metabolic markers in our analysis. In the revised manuscript, we have acknowledged the omission of key metabolic markers such as HbA1c and insulin levels due to data limitations. We have added a statement in the Discussion section to clarify that these variables were not included in the analysis, which may limit the comprehensive adjustment of confounders. Additionally, we have emphasized our plan to collect these important metabolic parameters in future studies to further improve the predictive model.

Finally, some relevant metabolic indicators, such as glycated hemoglobin (HbA1c) and direct measures of insulin resistance, were not included due to data limitations, which may affect the comprehensive adjustment of confounders and the model’s predictive accuracy, and we plan to collect these important metabolic parameters to further improve the predictive model in the future.(View in Limitation of Discussion Section )

4. Depth of Discussion
   Strengthen the Discussion by addressing the clinical implications and translational significance of the findings. Examples include:
   - Is the TyG-BMI cutoff applicable to other ethnicities or regions?
   - How can this tool be integrated into existing NAFLD screening workflows (e.g., as a replacement for or complement to ultrasound)?
   Answer: Thank you for the insightful suggestion. We have revised the Discussion to highlight the clinical relevance of TyG-BMI. As a simple, non-invasive, and cost-effective tool based on routine clinical data, TyG-BMI may complement or triage NAFLD screening in primary care, especially where imaging resources are limited. In our cohort, a TyG-BMI ≥251.741 showed high specificity (91.5%) for identifying high-risk individuals, while <212.886 showed high sensitivity (80.0%) for ruling out NAFLD. Although derived from a Chinese elderly diabetic population, similar values reported in other studies suggest broader applicability, though further validation across ethnicities is needed. Integration into EHR systems may support early detection and monitoring in clinical workflows.(View in Discussion Section in Iine 291-295)

5. Enrichment of Conclusions
Emphasize scenarios where the TyG-BMI index holds particular promise for implementation, such as primary care screening, to underscore its clinical relevance.

Answer: In response, we have added a clarification highlighted in Line 304-308:”In conclusion, although the prevalence of NAFLD remained high among elderly individuals with diabetes, a stepwise decline was observed with increasing age in both elderly males and females. In this population, the TyG-BMI index demonstrated superior predictive accuracy for NAFLD compared to the TyG index alone. Given its simplicity, non-invasiveness, and low cost, the TyG-BMI index holds particular promise for implementation in primary care settings as a practical screening tool to identify elderly diabetic patients at high risk for NAFLD, thereby underscoring its clinical utility in early detection and prevention strategies.”

6. Ethics Statement Clarification
The ethics declaration cites "previous data and cross-sectional design" to justify exemption from informed consent. However, the degree of data anonymization and original consent scope for future research use remain unclear. It is recommended to supplement whether the data is completely anonymous (cannot be traced back to individuals), and to confirm whether the original data collection included consent clauses for future research purposes.

Answer: In the revised manuscript , we have already made modifications in Line 67-73 to address the Ethics Statement clarification.

7.English Grammar
Enhance grammatical accuracy throughout the manuscript. For example, the lines 28-29 in the abstract (The AUCs of TyG and TyG-BMI indicated that both had predictive value for NAFLD, with TyG-BMI showing the highest predictive accuracy) contains a superlative error. As only two indices are compared, "higher" is more appropriate than "highest."

Answer: Thank you for the reviewer’s suggestion. The use of "highest" in the original manuscript was inappropriate due to our oversight, and it has been corrected to "higher" in Line 28.

Reviewer #3: This study investigates the predictive value of two indices (TyG and TyG-BMI) for non-alcoholic fatty liver disease (NAFLD) in elderly diabetic patients. The topic is timely and relevant, especially given the increasing prevalence of NAFLD and metabolic syndrome in aging populations. However, the manuscript would benefit from improvements in clarity and scientific rigor in several sections. Below are my comments.

1. **Figures and tables should be more clearly labeled. For instance, define all abbreviations in each figure/table legend. The logistic regression models need more detailed reporting—specifically about multicollinearity, confounding variables, and model fit. ROC curves and AUCs are presented but should be accompanied by confidence intervals and statistical comparisons (e.g., DeLong test).**

Answer: Thank you for this helpful suggestion. In accordance with your suggestions, we have implemented the following revisions:

(1) We have revised all figure and table legends to include definitions of all abbreviations (View in Tables and figures)

(2) We have added a section to the Results (or Methods) section detailing the assessment of multicollinearity, including Variance Inflation Factors (VIFs) for all predictor variables. In addition, we have included additional measures of model fit (Akaike Information Criterion) to better demonstrate the adequacy of the model. Specifically as follows:

*Utilizing the Akaike Information Criterion (AIC) to assess the relative fit of competing models, a lower AIC value indicates a superior model in terms of parsimony and explanatory power. Variance Inflation Factors (VIFs) were calculated to assess multicollinearity among the independent variables in the model, and a VIF greater than 5 is commonly considered indicative of high multicollinearity.(View in Statistical analysis of Methods section)*

*Otherwise, the decreasing AIC values across the models suggest a progressively improved model fit, indicating a better balance between model complexity and explanatory power for the observed data (Table 2). A VIF value of less than 5 for each variable in the model suggests that multicollinearity is not a concern(Supplemental Table 1) (View in ‘****Association of TyG and TyG-BMI with NAFLD in elderly participants with diabetes’ of Results section****)*

(3) The previous version already included confidence intervals for the AUCs and statistical comparisons. To enhance clarity, we have added corresponding annotations to the table 4. The specific revisions are as follows:

*The predictive abilities of TyG and TyG-BMI for NAFLD were evaluated using the area under the receiver operating characteristic (AUROC) curves. The AUROC values were compared using the Delong test.(View in Statistical analysis of Methods section)*

**Table 4 The optimal cutoff point for the triglyceride-glucose-body mass index and triglyceride-glucose in diagnosing non-alcoholic fatty liver disease in training and validation group.**

| Dataset | Marker | CutOff | AUC (95%CI) | P value^*^ | Sensitivity | Specificity | PPV | NPV |
| --- | --- | --- | --- | --- | --- | --- | --- | --- |
| Training | TyG-BMI | 219.028 | 0.771(0.758-0.784) | Reference | 0.748 | 0.657 | 0.649 | 0.754 |
|  | TyG | 8.929 | 0.678(0.663-0.692) | <0.001 | 0.690 | 0.585 | 0.585 | 0.690 |
| Validation | TyG-BMI | 215.712 | 0.756(0.738-0.779) | Reference | 0.770 | 0.626 | 0.641 | 0.759 |
|  | TyG | 9.109 | 0.703(0.680-0.725) | <0.001 | 0.586 | 0.723 | 0.646 | 0.668 |

AUC, area under curve; PPV, positive predictive value; NPV, negative predictive value; TyG-BMI, triglyceride-glucose-body mass index; TyG, triglyceride-glucose.

* P value for DeLong test

Ultrasound is commonly used to detect fatty liver but may not reliably identify mild steatosis. Is it accurate enough to diagnose NAFLD in this study population?
Answer: In accordance with the suggestion from the senior reviewer, we have revised the original manuscript accordingly. We added the following statement in Line 296-298 :”While ultrasonography is widely used for NAFLD detection in clinical practice, its limited sensitivity, particularly in cases with mild steatosis, may lead to underdiagnosis. This may introduce selection or information bias and affect the study's accuracy and reliability”.

Several types of diabetes medications have shown promise in improving NAFLD, particularly for those with type 2 diabetes. Pioglitazone stands out as a well-studied option, with strong evidence of improvement in liver histology, especially in patients with biopsy-proven NASH. Additionally, GLP-1RAs and SGLT2is have also demonstrated beneficial effects on NAFLD, including potential reductions in liver fat and improved liver enzymes. Have the authors reported the medication histories of all participants?

Answer: Thank you for raising this important question. We fully acknowledge that diabetes medications (such as pioglitazone, GLP-1 receptor agonists, and SGLT2 inhibitors) may have significant effects on liver histology and biochemical markers in NAFLD/NASH patients.

In the current study, we did collect medication histories for some participants but have not yet conducted a comprehensive analysis of all patients' diabetes drug usage. We recognize that this information is crucial for interpreting the study results. Therefore, in our next steps, we will:

1. Systematically collect and analyze detailed medication histories for all participants, particularly focusing on diabetes-related drugs (e.g., pioglitazone, GLP-1RAs, SGLT2 inhibitors);

2. Evaluate the effects of these medications on metabolic parameters, liver fat content, and liver enzyme levels in NAFLD/NASH patients;

3. Adjust for medication history in statistical analyses to more accurately assess the impact of other factors (e.g., genetics, lifestyle) on disease progression.

We believe these additional analyses will further enhance the comprehensiveness and reliability of our study.

As noted in the discussion, “The TyG-BMI index, developed by Wang et al. in a Japanese population, has been shown to predict NAFLD effectively.” However, this study focuses on elderly patients with diabetes. It is unclear what new insights this research contributes beyond existing literature on TyG indices and NAFLD.

The phrase “Error! Reference source not found” appears multiple times in the manuscript. Please review the document carefully and correct these errors.

Answer: Thanks for your constructive reminder. As we clarified in the manuscript, although the prevalence of NAFLD remained high among elderly individuals with diabetes, a stepwise decline was observed with increasing age in both elderly males and females. It suggested that the disease may exhibit distinct clinical features or risk profiles in elderly individuals compared to younger adults. In addition, NAFLD may be associated with IR in diabetes patients. We confirmed that the TyG-BMI index could demonstrate excellent predictive accuracy for NAFLD on elderly patients with diabetes.

Revised the phrase "Error! Reference source not found".
