# Supplementary material for: Comparison of the triglyceride-glucose index and triglyceride-glucose-body mass index for predicting non-alcoholic fatty liver disease in elderly diabetic patients
Source: PLoS One. 2026 Feb 2;21(2):e0341109. doi: 10.1371/journal.pone.0341109 (PMC12863506; doi:10.1371/journal.pone.0341109)
Supplement: Attachment — Submitted filename: Review- NAFLD in elderly in DM.doc [file pone.0341109.s006.doc]

Review- Comparison of the triglyceride-glucose index and triglyceride-glucose-body mass index for predicting non-alcoholic fatty liver disease in elderly diabetic patients.

Revised manuscript with changes - review

1) Abstract: Line 17:

Developing an effective, simple, noninvasive method to

identify NAFLD, track disease progression, and monitor treatment effects is importance- correction – important

Line 17 and 18: This study aims to assess the value of triglyceride-glucose index (TyG) and triglyceride-glucose-body mass index (TyG-BMI) in detecting NAFLD elderly patients with diabetes mellitus (DM). correction to - NAFLD in elderly patients.

2) Table 1- suggest change of Nomenclature- use presence and absence of NAFLD- use of non-NAFLD is confusing and almost represents as Alcoholic fatty liver disease.
3) Reference sources needed- showing as errors?- need explanation as to why they are still being used that way
