# Supplementary material for: Comparison of the triglyceride-glucose index and triglyceride-glucose-body mass index for predicting non-alcoholic fatty liver disease in elderly diabetic patients
Source: PLoS One. 2026 Feb 2;21(2):e0341109. doi: 10.1371/journal.pone.0341109 (PMC12863506; doi:10.1371/journal.pone.0341109)
Supplement: Attachment — Submitted filename: Response_to_Reviewers_auresp_2.docx [file pone.0341109.s008.docx]

**Reviewer 4**

**Reviewer 5:**

The results part requires and in depth work to constrictively present the results and the dedicated references, and to allow targeted comments for reviewing such as a comment on the fact that NAFLD decreases gradually as subjects aged, or why the three different models authors have used are not subsequently studied. The layout should be also redone. The quality of the figures should be improved.

Answer: Thanks for your careful advice. We submitted the Supplemental Table S1 to show the relationship between the prevalence of NAFLD and age. And due to the length limitation and research objective of the paper, there was no further analysis on the low incidence rate of NAFLD among the elderly. In future, we will conduct the epidemiologic study of NAFLD in the eldly. And we have added the relevant content at the end of the Discussion Part in Line 302-303. Furthermore, the three models just proved that adjusting different types of variables could make the model stable, so we did not conduct further analysis and research. We have adjusted the position of Table 2 in Line 156. And We have uploaded the clear figures again.

**Reviewer 6:**

1) Abstract: Line 17:

Developing an effective, simple, noninvasive method to identify NAFLD, track disease progression, and monitor treatment effects is importance- correction – important.

Line 17 and 18: This study aims to assess the value of triglyceride-glucose index (TyG) and triglyceride-glucose-body mass index (TyG-BMI) in detecting NAFLD elderly patients with diabetes mellitus (DM). correction to - NAFLD in elderly patients.

Answer: Thanks for your kind reminder. “NAFLD in elderly diabetic patients” is more suitable in this part. We have changed this content in Line 17.

2) Table 1- suggest change of Nomenclature- use presence and absence of NAFLD- use of non-NAFLD is confusing and almost represents as Alcoholic fatty liver disease.

Answer: Thank you for your careful reminder. We have changed the Table title to “Baseline characteristics for the training and validation groups by NAFLD or non-NAFLD status in diabetes population.”

3) Reference sources needed- showing as errors?- need explanation as to why they are still being used that way.

Answer: Thank you for pointing out our mistakes. Due to unfamiliarity with the literature citation software, there were some errors in the manuscript. We have double checked and resolved them.
