# Supplementary material for: Comparison of the triglyceride-glucose index and triglyceride-glucose-body mass index for predicting non-alcoholic fatty liver disease in elderly diabetic patients
Source: PLoS One. 2026 Feb 2;21(2):e0341109. doi: 10.1371/journal.pone.0341109 (PMC12863506; doi:10.1371/journal.pone.0341109)
Supplement: Attachment — Submitted filename: Response_to_Reviewers_auresp_3.docx [file pone.0341109.s009.docx]

**Reviewer 5**:

Zhu et al. sent a manuscript entitled « Comparison of the triglyceride-glucose index and triglyceride-glucose-body mass index for predicting non-alcoholic fatty liver disease in elderly diabetic patients», for a second round of a reviewing process to Pone.

The aim of this study was to highlight a prospective and non invasive tool, the triglyceride-glucose-body mass index (TyG-BMI), to predict non-alcoholic fatty liver disease with the help of a large cohort of 6,882 individuals aged at least 60 years old and with diabetes mellitus.

The introduction and the methodology part are still good and clearly present the subject with the dedicated references.

But, the results part remains insufficient and requires and in depth work to constructively present the results and the dedicated references, and to allow targeted comments for reviewing such as a comments on the fact that NAFLD seems to decrease gradually as subjects aged, or why the three different models authors have used are not subsequently studied. In addition for this last point, authors should justify the choice of the parameters in each model (with the dedicated references). This lack of organisation could be easily put right and might strengthen the impact of the results of the authors.

Line 85 Error reference

Order of the figures

Table 1, authors should emphasize the differences plotted between the two groups.

Line 152 and 154 layout

Line 183 layout Figure title

Line 216 and 268 « cutoff »

LIne 282 de novo « italic »

The quality of the figures should be improved.

**Answer:**

Thanks for your careful advice. We have a comment about the fact that NAFLD seems to decrease gradually as subjects aged in Line 240-246 and we will analyze the impact of age on NAFLD in the elderly in future research due to the length limitation and research direction of this paper. The three models with the decreasing AIC values and a VIF value of less than 5 were used to prove that adjusting different types of variables could make the model stable, so we did not conduct further studies. As to the choice of the parameters in each model, we selected only those parameters that demonstrated statistical significance in univariate analysis based on the literature (Wang R, Dai L, Zhong Y, et al. Usefulness of the triglyceride glucose-body mass index in evaluating nonalcoholic fatty liver disease: insights from a general population. Lipids Health Dis, 2021, 20(1): 77) and then divided them into general demographic data and the general examination items in our hospital, like complete blood count and liver-kidney glyco-lipid test. We also modify the annotation below Table 2 in Line 157-159 to more clearly indicate the adjustment of each model variable.

At last, we modify the “Error reference” in Line 85, change the order of the figures and figure title in Line 86, 176, 184 and 235. Apart from LDL-C, there are no significant differences between the training and validation groups, which indicates that the data has the same distribution between the training and validation groups to achieve more stable and accurate results later. We separately expressed the significant differences within the training and validation sets in Line 141-145. We also changed the “cutoff” and “de novo” with the correct format.

The quality of the figures has been reuploaded, and we found that the higher clarity of the figures need to be downloaded instead of in submission view. So we uploaded the PDF and TIFF format together.
