# Supplementary material for: Comparison of the triglyceride-glucose index and triglyceride-glucose-body mass index for predicting non-alcoholic fatty liver disease in elderly diabetic patients
Source: PLoS One. 2026 Feb 2;21(2):e0341109. doi: 10.1371/journal.pone.0341109 (PMC12863506; doi:10.1371/journal.pone.0341109)
Supplement: Attachment — Submitted filename: Response to Reviewers20251214.docx [file pone.0341109.s010.docx]

**Reviewer 5**:

Zhu et al. sent a manuscript entitled « Comparison of the triglyceride-glucose index and triglyceride-glucose-body mass index for predicting non-alcoholic fatty liver disease in elderly diabetic patients», for a second round of a reviewing process to Pone.

The aim of this study was to highlight a prospective and non invasive tool, the triglyceride-glucose-body mass index (TyG-BMI), to predict non-alcoholic fatty liver disease with the help of a large cohort of 6,882 individuals aged at least 60 years old and with diabetes mellitus. The introduction and the methodology part are still good and clearly present the subject with the dedicated references.

As mentioned previously, the description of the results present a lack a clarity, and still require an in depth re-organisation and presentation.

Some additional typo comments

Line 27/81 remove « space »

Line 86

Cut-off or cutoff

Line 230 Fig.4 —> Figure 4

Line 235 FLD —> NAFLD

Line 261, 264, 265 et al. (Italic)

**Answer:**

We sincerely thank the reviewer for these insightful and constructive comments, which have substantially improved the scientific rigor and clarity of our manuscript. We have carefully addressed each concern raised, and the specific revisions are detailed below:

(1) Explanation for age-related decline in NAFLD prevalence

We thank the reviewer for highlighting this important finding. This finding can be attributed to the age-related physiological changes or the influence of survivor bias. We have added a dedicated discussion of this phenomenon in the Discussion section, Lines 267-270.

(2) Justification for the three hierarchical models with dedicated references

We fully agree with the reviewer's concern. The covariates included in our three hierarchical models were selected systematically based on established literature and prior evidence of their associations with NAFLD. The AIC values were used to compare the relative fit of the three models and to determine which set of covariates provided the optimal balance between explanatory power. Additionally, it should be noted that the predictive ability and reclassification statistics, as well as the clinical utility analysis, were all performed specifically for the optimal predictive models (Model 3) with TyG/TyG-BMI according to AIC values. We have substantially revised the Methods section to provide a comprehensive justification for our hierarchical modeling strategy, supported by dedicated references. We made the following revisions in the Methods, Line 109-122 and Results sections, Line 161-162.

This stepwise approach allows readers to assess the independence and robustness of the associations across different levels of covariate adjustment, which is a recommended practice in epidemiological research.

(3) Enhanced presentation and justification of subgroup analyses

We appreciate this comment and have substantially enhanced the Results section to provide more detailed justification and clearer presentation of our stratified analyses. We have implemented corresponding revisions in the Methods, Line 125-127, Results, Line 174-178 and Table 3, Line 182-185.

(4) Modification of some additional errors

We are grateful for the careful advice. We remove the « space » in Line 27 and 81. The “Fig. 4” has been revised as “Figure 4” in Line 244. We modify the caption and legend of Fig. 4 in Line 250-252 and change the corresponding content mentioned above. At last, we change all instances of “et al.” to italics.
